# Exploring the potential of public proteomics data

**DOI:** 10.1002/pmic.201500295

**Published:** 2015-12-15

**Authors:** Marc Vaudel, Kenneth Verheggen, Attila Csordas, Helge Ræder, Frode S. Berven, Lennart Martens, Juan A. Vizcaíno, Harald Barsnes

**Affiliations:** ^1^ Proteomics UnitDepartment of BiomedicineUniversity of BergenBergenNorway; ^2^ Medical Biotechnology CenterVIBGhentBelgium; ^3^ Department of BiochemistryGhent UniversityGhentBelgium; ^4^ Bioinformatics Institute GhentGhent UniversityGhentBelgium; ^5^ European Molecular Biology LaboratoryEuropean Bioinformatics Institute (EMBL‐EBI)Wellcome Trust Genome CampusHinxtonCambridgeUK; ^6^ Department of Clinical ScienceKG Jebsen Center for Diabetes ResearchUniversity of BergenBergenNorway; ^7^ Department of Clinical MedicineKG Jebsen Centre for Multiple Sclerosis ResearchUniversity of BergenBergenNorway

**Keywords:** Bioinformatics, Computational proteomics, Data analysis, Databases, Data standards

## Abstract

In a global effort for scientific transparency, it has become feasible and good practice to share experimental data supporting novel findings. Consequently, the amount of publicly available MS‐based proteomics data has grown substantially in recent years. With some notable exceptions, this extensive material has however largely been left untouched. The time has now come for the proteomics community to utilize this potential gold mine for new discoveries, and uncover its untapped potential. In this review, we provide a brief history of the sharing of proteomics data, showing ways in which publicly available proteomics data are already being (re‐)used, and outline potential future opportunities based on four different usage types: use, reuse, reprocess, and repurpose. We thus aim to assist the proteomics community in stepping up to the challenge, and to make the most of the rapidly increasing amount of public proteomics data.

**Abbreviation**
PSM: peptide to spectrum match

## 1 Introduction

### 1.1 Background

**Figure 1:**
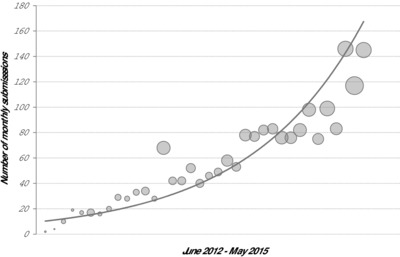
The amount of publicly available proteomics data is increasing, here indicated by the monthly submission statistics for PRIDE from June 2012 to May 2015. The *x*‐axis represents the months and the *y*‐axis the monthly number of submissions. The size of the bubbles indicate the data amount submitted each month. Note that the cumulative size of PRIDE data reached the 100 TB milestone in April 2015.

Historically, a large proportion of the proteomics community was reticent to openly share the data they produced. However, the sharing of not only the knowledge obtained through proteomics experiments (through scientific publications), but also of the underlying data, has increasingly become standard practice, and is now even mandatory or strongly advised in many of the relevant scientific journals 1, 2, 3. In addition, a number of funders (e.g. the Wellcome Trust and the NIH) are enforcing the public deposition of data from projects they fund as a way to maximize the value of the funds provided. As a result, the amount of publicly shared MS‐based proteomics data has grown substantially, both in terms of number of submission and total data amount, as illustrated in Fig. 1.

Two key factors strongly contributed to this success: first, the sharing of the data has become much easier with the development of user‐friendly tools and infrastructure; and second, the proteomics community, triggered by scientific journals and funders, has now agreed that it is good scientific practice to make the underlying data available when publishing novel findings.

**Figure 2:**
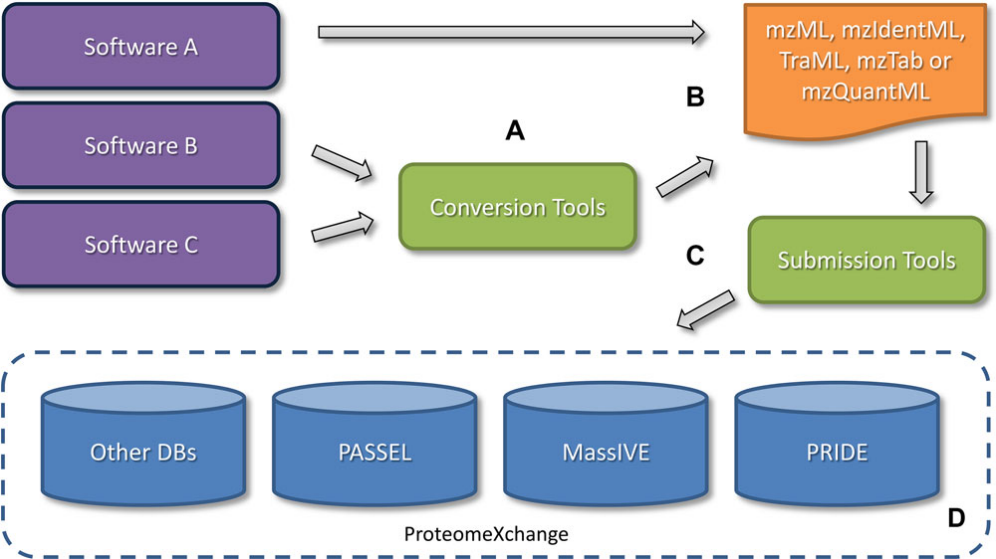
The major milestones that enabled efficient proteomics data sharing: (A) standard data formats for sharing proteomics data, (B) data format converters and software exporters able to generate output in the standard formats, (C) tools for simplifying the submission of proteomics data to central proteomics repositories, and (D) central proteomics repositories that store and disseminate public proteomics data, here indicated by the main ProteomeXchange member repositories.

There were several challenges to overcome in order to get to this point, see Fig. 2. The first of these challenges was the need for central and long‐term public repositories to store the generated data. Several such generic repositories are now available, for example PRIDE 4, GPMDB 5, PeptideAtlas 6, and MassIVE (http://massive.ucsd.edu/ProteoSAFe) for shotgun results; and PASSEL 7, SRMAtlas (http://www.srmatlas.org), and Panorama 8 for targeted proteomics quantification data. More specific databases have also been established, related to: diseases, for example TBDB for tuberculosis 9; organisms, for example ProteomicsDB 10 and the Human Proteome Map 11 for the human proteome, and pep2pro for *Arabidopsis* 12; or subproteomes, for example CSF‐PR 13 for cerebrospinal fluid or TOPPR 14 and TopFIND 15 for in vivo cleaved proteins. For a comprehensive overview of the current proteomics databases and repositories, please see Perez‐Riverol et al. 16.

The next milestone was the development of data‐sharing standards and associated software libraries, allowing ready access to otherwise proprietary data formats 17. This ongoing endeavor, led by the HUPO‐PSI (Human Proteome Organization−Proteomics Standards Initiative−http://www.psidev.info), has resulted in key data standards for the field, including mzML (for MS data), mzIdentML (for peptide/protein identification data), mzTab (for peptide/protein identification and quantification data), mzQuantML (for peptide/protein quantification data), and TraML (for transition lists in targeted proteomics approaches) 18, 19, 20, 21, 22. Importantly, support for these standards is provided through software libraries or tools such as ProteoWizard 23, PRIDE Converter 24, 25, mzidLibrary 26, and PRIDE Inspector 27. Successful adoption of these standards is moreover demonstrated by the existence of import and/or export capabilities in many of the most popular software in the field.

The final piece of the puzzle was the creation of an overarching system to share submitted data between repositories, and to develop a single, user‐friendly submission workflow. This goal was obtained with the establishment of the ProteomeXchange consortium 28, which connects some of the most used proteomics databases (at present PRIDE, MassIVE, PASSEL, and PeptideAtlas) with a single submission system and the use of unique identifiers that can be tracked across these databases and over time.

However, while publicly available proteomics data represent an invaluable resource for extracting new knowledge 29, they have so far, with a few notable exceptions, remained largely unused. At the same time, data reprocessing has become the standard in related fields, such as genomics, see Rung et al. 30. The time has now come for the field of proteomics to also start utilizing its public data as a test bed for novel ways of interpreting proteomics data, and as a potential goldmine for new discoveries. The heterogeneous nature of the accumulated data also provides a global view on the state of the art and the evolution of the field as a whole, and reduces bias toward specific protocols or instruments.

**Figure 3:**
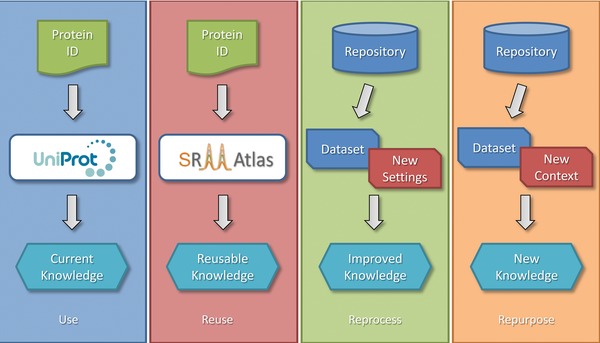
The four ways in which public proteomics data can be utilized: (i) use, (ii) reuse, (iii) reprocess, and (iv) repurpose. See main text for details.

There are four ways in which these shared proteomics data can be utilized: (i) *use*, (ii) *reuse*, (iii) *reprocess*, and (iv) *repurpose* (Fig. 3), each of which will be described in detail in the following sections.

### 1.2 Data use through protein knowledge‐bases

An example of the direct *use* of proteomics data is by looking up information about a given protein as indexed in an online protein knowledge‐base, such as UniProt 31 or neXtProt 32. This does not result in knowledge beyond what has already been published, but does provide the means to understand the current context of the protein(s) in question. For example, MS proteomics data deposited in public repositories is used by UniProt and neXtProt to enrich sequence annotations at the level of the evidence that supports protein existence (isoforms and variant sequences included). This information is provided to users in two ways: (i) via the protein evidence values, or (ii) through cross‐references to proteomics resources (e.g. PRIDE and PeptideAtlas, among others). The next step will be the incorporation of PTMs based on the information available in proteomics repositories, as is already done in databases such as PhosphoSitePlus 33. Currently, the main integration of this information occurs via manual curation of relevant publications.

### 1.3 Reusing data to improve proteomics approaches

In the case of *reuse*, information is not only extracted, but also reused in new experiments with the potential of generating new knowledge. One of the best examples is the reuse of SRM transitions generated by others, via SRMAtlas (http://www.srmatlas.org) or Panorama 8, where existing transitions for specific proteins in a given instrumental setup can be found. Note that it is also possible to develop tools to look for novel transitions in publicly available shotgun datasets. For example, MRMaid 34, PeptidePicker 35, 36, and ProteomicsDB 10 do this by reusing identification data coming from PRIDE and other sources.

One particular type of data reuse, already popular in other disciplines, is to analyze data from a large number of publications/datasets in a combined way, so‐called *meta*‐analysis studies. Indeed, the availability of large amounts of proteomics data has the advantage that it can be used for data mining purposes, that is extracting aggregated knowledge from the data provided by the community worldwide. The principle being: the more data, the better the understanding.

In fact, *meta*‐analysis studies can indeed provide new information that can be directly applied in proteomic analytic workflows. One example is a study aimed at improving the understanding of the cleavage mechanism and performance of trypsin 37, 38, a crucial parameter in proteomic workflows. By inspecting the cleavage profile of all peptide identifications deposited in PRIDE, it was possible to train an algorithm that predicts trypsin cleavage sites, a functionality that is available through a web interface 39. Similarly, the study of deposited data was used to monitor peptide elution during LC, and enabled the optimization of gradients in silico 40. Public MS data have also been mined to study the fragmentation pattern of different fragmentation methods 41, and to predict peptide fragmentation patterns 42.

PRIDE data have also been reused through the combination of data from significantly different experimental setups. For example, Klie et al. 43 used a noise‐tolerant algorithm to extract new knowledge from the datasets that comprise the HUPO Plasma Proteome Project 44. Another example can be found in Müller et al. 45, where two proteomics datasets related to the CNS were remapped against a more recent version of the protein sequence database used in the original studies. This enabled the authors to look for the expression of specific splice isoforms from CNS‐related genes. Finally, in another example of PRIDE data reuse, UniProtKB was determined to be the most suitable reference database for long‐term proteomics data storage 46.

Large‐scale biological results can also be reused because of their indexing in databases, notably via so‐called BioMarts 47 or more recently, web services 48, 49. Mining such data in their biological context may allow the extraction of novel biomarkers, as discussed in Griss et al. 50.

### 1.4 Reusing data via spectral libraries and spectral archives

Additional spectrum interpretation strategies such as de novo sequencing or spectral databases are also promising approaches to increase the identification rate of spectra in MS‐based proteomics. The creation of spectral libraries most strongly benefits from the growing amount of shared data 51, 52. Several repositories, including PeptideAtlas, GPMDB, and PRIDE, and research groups such as the one at NIST (National Institute of Standards and Technology), provide spectral libraries for different species, which can in turn be used to perform spectral searches.

When assessing the similarity of spectra, spectral clustering can be performed 53, 54, 55, 56. While transitive identifications and consensus or representative spectra have been reported in all of these studies, the concept was further developed in the creation of spectral archives 57. Spectral clustering has since been adopted by PRIDE to make quality assessments on the submitted data at the peptide to spectrum match (PSM) level 58. After clustering, a representative spectrum is built for all peptides consistently identified across different datasets. The accuracy of this representative spectrum thus improves with every new dataset submitted to PRIDE, allowing an automated quality assessment of the PSM data. The key role of proteomics repositories in the further development of spectral archives was highlighted by H. Lam, who envisioned a future where it would be possible to perform a centralized data analysis by performing spectral searches 59.

### 1.5 Data reprocessing through improved bioinformatic approaches

In the case of *reprocess*, the data are reprocessed with the intention of obtaining new knowledge or to provide an updated view on the results. This can result in novel findings, but mainly serves the same purpose as the original experiment. For example, a shotgun dataset can be reprocessed with a different algorithm or an updated sequence database.

Perhaps, the simplest step one can take when reprocessing a dataset is to analyze the potential effect of adding common contaminants if these were not included in the original search, as this makes it possible to rule out common false positive findings. For example, it could potentially turn out that an important finding could be better explained by a match with a common contaminant such as human keratin or trypsin 60. For instance, a standard list of contaminants can be found in the common Repository of Adventitious Proteins (cRAP—http://www.thegpm.org/crap), provided by the GPM team.

The gene and protein sequence databases that identification depends on are constantly evolving and improving 46. This means that reprocessing a proteomics dataset with an updated version of the gene or protein database can result in improved findings. This is particularly true for poorly annotated species. In addition, updating a database to include known isoforms and/or mutations will provide a different view of the dataset.

Analogously, the software used to process proteomics data is also constantly improving, either by the further development of existing algorithms or by the establishment of new analysis approaches. The use of up‐to‐date techniques for the reprocessing of older datasets allows valuable information to be extracted from the acquired data without the need to repeat the experiment. This is particularly important for data from valuable or unique samples, where it ensures that as much information as possible can be obtained from these samples.

It should be noted that some of the existing proteomics databases, most notably GPMDB 5 and PeptideAtlas 6, routinely reprocess their data using dedicated bioinformatics tools and pipelines. GPMDB makes use of the X!Tandem search engine 61, whereas PeptideAtlas employs the Trans Proteomic Pipeline 62. The data reprocessed by PeptideAtlas is organized into different builds, each including data from a single proteome (e.g. human) or subproteome (e.g. human plasma). Each build is generated based on the raw MS/MS spectra submitted to PeptideAtlas over the years, or from data deposited in other public repositories, for example PRIDE. In addition to human, many species now have specific PeptideAtlas builds, including, for example *Candida albicans* 63 and horse 64, among many others.

The GPMDB pipeline reprocesses the MS/MS data provided by users or raw data stored in other repositories, such as those from ProteomeXchange. Till the end of 2014, some of the reprocessed datasets were highlighted on a weekly basis on the GPM website (http://www.thegpm.org/news.html).

Both resources, PeptideAtlas and GPMDB, are also joining efforts in the context of the Chromosome‐based (C‐) and Biology/Disease (B/D) Human Proteome Projects (HPP) 65, 66, together with neXtProt and the antibody‐based resource Human Proteome Atlas 67. This is a clear example of the utility of large‐scale and centralized (re‐)processing, as it can ensure consistent processing and thus comparable results. The C‐HPP team provides regular updates on the status of completion of the human proteome and on the enumeration of the so‐called “missing” proteins, that is proteins that have never been reliably detected experimentally 68.

### 1.6 Reusing and reprocessing enables scientific discussion

Perhaps, the most common current use case for shared proteomics data is the evaluation of existing results, often as part of the manuscript review process. This can be achieved by inspecting the data as provided by the authors, or by reprocessing the raw data by mimicking the original processing and then assessing the reproducibility of the results. The evaluation can be carried out at two levels: at the level of the individual PSMs, or at the level of the entire dataset. An example of the former is the checking of spectrum annotation quality, for example for post‐translationally modified peptides. This can, for example, be achieved via the use of visualization tools such as MS‐viewer 69, Scaffold Viewer, Thermo MSF Viewer, Peptizer 70, ProteoIDViewer 26, or TOPPView 71, among others.

For validation at the dataset level, tools such as PRIDE Inspector 27 and PeptideShaker 72, can be used to inspect and reprocess the data, respectively. Note that PeptideShaker provides a direct connection to PRIDE datasets to enable their streamlined reprocessing. The need for visual and interactive solutions should be noted here, as this can dramatically improve the validation procedure compared to looking at static images or tables 73.

One of the most famous examples of data discussion, involving both visual inspection and reprocessing, is related to the proteomics investigations of *Tyrannosaurus rex* fossil bone samples. The initial publications by Asara et al. 74, 75 proved controversial in the proteomics community (see, e.g. 76, 77). As a consequence, the authors decided to make their data publicly available (PRD000074 in PRIDE), such that other researchers could inspect and reprocess the data themselves. Among others, this resulted in Bern et al. concluding that the original data did not contain any *T. rex* proteins 78. The debate remains to be definitively settled, but the spirited scientific discussion highlights the importance of making the underlying data for published work available so that all sides can scientifically and reasonably discuss the findings based on the same evidence.

Another example is a study by Bromenshenk et al., which claimed to have found a link between viral and fungal contamination and the ongoing honey bee colony collapse disorder 79, a study that sparked global public interest. However, after the authors shared the data with others (available on request only), it became clear that this too could be a false positive outcome due to the systematic misidentification of bee‐derived spectra as viral or fungal sequences, due to searching against a protein sequence database that lacked all honey bee sequences 80, 81, 82. This discussion too still continues; however, as the same dataset was recently used to illustrate the opinion that, in order to improve statistical power, researchers should remove irrelevant peptides from the database before searching 83. Here again, the inspection and reprocessing of the original experimental data enabled a scientific discussion and made it possible to collectively improve the scientific output, and paved the way for new discoveries 84.

More recently, there is an ongoing debate about the two drafts of the human proteome published in *Nature* in 2014 10, 11. Both studies provided an exemplary precedent by sharing all generated data (available as datasets PXD000561 and PXD000865 in PRIDE). This has enabled the community to start a discussion about the reliability of the results, see for instance Ezkurdia et al. 85.

### 1.7 Data repurposing in proteogenomics studies

Finally, when *repurposing* public data, these data are considered in light of a question or a context that is entirely different from the original study. It should be noted that repurposing thus often involves reprocessing as well. One example is the reprocessing of proteomics datasets to improve genome annotation in so‐called proteogenomics approaches. For example, Brosch et al. reprocessed shotgun proteomics data from PeptideAtlas to discover novel protein‐coding genes and to improve gene annotation in the mouse genome 86. At the time, they found alternatively spliced translations from 53 genes along with ten entirely novel protein‐coding genes. Another example is provided by LNCipedia 87, a resource for human long noncoding RNAs. PRIDE‐based reanalysis of human proteomics data has provided evidence that some long‐noncoding RNAs in LNCipedia are potentially translated to proteins 87.

In another proteogenomics study, Ezkurdia et al. reprocessed public proteomics data available in GPMDB and PeptideAtlas to identify peptides covering 35% of the genes annotated by the GENCODE consortium for the human genome 88. Among other findings, they found that 150 genes expressed multiple alternative protein isoforms. Additionally, in a second analogous study, they concluded that the human proteome was composed of around 19 000 protein‐coding genes 89, a lower number by around 1000 genes than the canonical assumption. In a related recent third study, they also reused public proteomics data from the same resources to suggest that most genes had a single dominant isoform at the protein level 90.

Existing proteomics data can also be reused in proteogenomics approaches. In a recent study devoted to psoriasis 91, the generated data were integrated with public data available in PRIDE (dataset PRD000053), proteomics data from other studies, and gene expression data available in the GEO (Gene Expression Omnibus) database 92. As a final example in this section, Zhu et al. employed public proteomics data to develop a tool that can identify differentially regulated splice variants 93.

Because of the massive amounts of publicly available data and their inherent heterogeneity, the chances of reliably detecting protein expression evidence is higher in such reprocessing and repurposing approaches. However, due to the unconventional sequence population of the databases in proteogenomics, and their often extensive size, the estimation of false positive rates by traditional approaches can be impaired 83, 94. In the near future, it is therefore expected that the creation of such sequence databases will be coupled to ribosome profiling data, to discern the exact start of translation of putative proteins 95. Indeed, tools such as ProteoFormer can already be used to generate proteomics‐compatible protein sequence databases from such ribosome profiling data 96.

### 1.8 Reprocessing for better PTM localization and repurposing to find new PTMs

Finding and localizing PTMs are essential tasks in proteomics data analysis 97, and for this purpose multiple PTM localization scores have been developed 98, for example A‐score 99, PTM score 100, MD‐score 101, phosphoRS 102, and D‐score 103. Setting a threshold for these scores is, however, challenging, and solutions have only recently been established 104, 105. If such approaches were not applied in the original analysis, it is worth reprocessing the data, as this can dramatically improve the quality of the PTM annotation on the protein sequences. The reported location of specific PTMs can furthermore be refined using additional techniques, for example by considering the three‐dimensional structure of the protein as indicated by Vandermarliere et al. 106.

It is also possible to repurpose existing datasets to look for PTMs that were not considered in the original analysis, for example via mass‐tolerant database searches 107. This task is made difficult by the substoichiometric nature of modified proteins, thus usually requiring experimental enrichment techniques to enable detection 108, 109, 110. It is therefore often not straightforward to simply reprocess a dataset to find such modifications, but here again, the large amount of public data increases the probability to uncover modified peptides. Successful studies have therefore used enriched phospho‐proteomics datasets to find peptides with unusual modifications that had a high probability of being co‐enriched. Matic et al. 111 reanalyzed a mouse dataset to identify a total of 88 mono–ADP‐ribosylation sites in 79 different proteins, with eight sites found modified also by ribose phosphate, a modification derived from ADP‐ribose. In the reanalysis of another mouse dataset, Hahne and Küster 112 discovered an O‐GlcNAc‐6‐phosphate modification on 23 peptides corresponding to 11 proteins.

### 1.9 Toward quantitative, across‐source reprocessing

At the moment few repositories contain quantitative proteomics data, though it is possible to include quantitative information in data submissions to proteomics resources such as PRIDE. However, it is not yet possible to visualize and inspect this information properly due to a lack of suitable tools. Such tool development will most likely hinge on more widespread adoption of the PSI standards for quantitative information, namely mzQuantML and mzTab.

There are, however, several protein expression databases, most notably MOPED 113 and PaxDb 114, which can be used to extract information about the expression levels of individual proteins. Both resources routinely make use of publicly available data in PRIDE and PeptideAtlas, among others. In PaxDb, identification data from filtered datasets are first mapped onto a common namespace, and quantification values are then derived after reprocessing with a standardized spectral counting pipeline. PaxDb is a meta‐resource in which protein expression is estimated across a number of species (more than 50 at the time of writing), and recently even across cell lines 115. MOPED presents a multiomics resource for human and model organisms, including at present gene, protein, and pathway expression information 116.

Another resource to highlight in this context is ProteomicsDB, which provides abundance estimates according to the label‐free intensity‐based iBAQ method 117. ProteomicsDB is one of the main outputs of the draft human proteome by Wilhelm et al. 10, and represents a nice example of data reprocessing. For their analysis, they combined their own generated experimental results with publicly available data. In fact, around 40% of the data used to generate this draft of the human proteome were obtained from public resources such as PRIDE, MassIVE, and PeptideAtlas (see Supporting Information Table 1 in 10 for the complete list). However, new datasets are reprocessed regularly and incorporated into ProteomicsDB, including also RNAseq data and phospho‐proteomics experiments.

**Figure 4:**
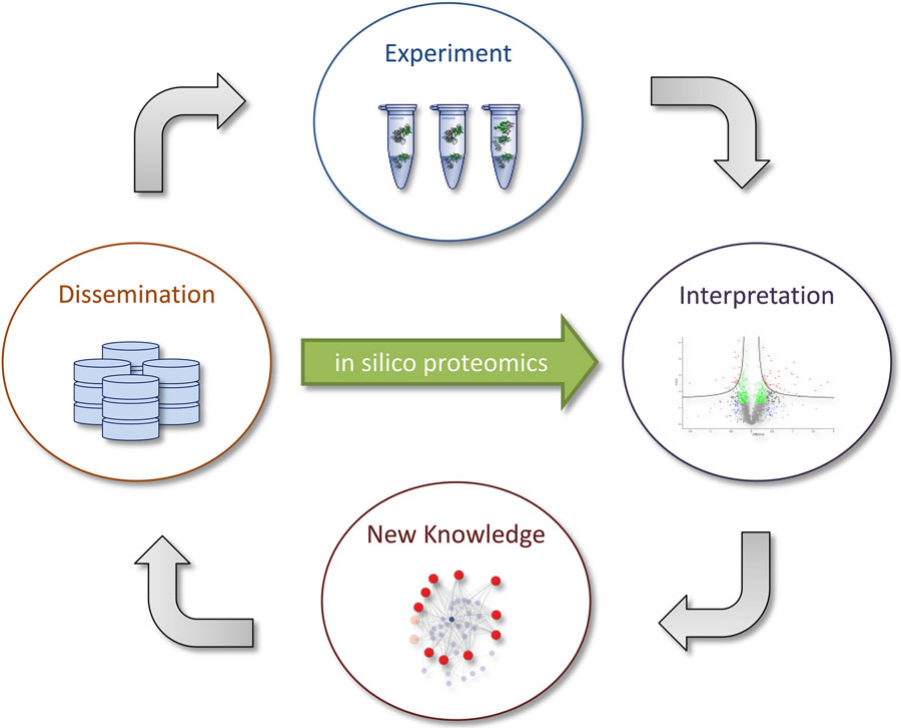
The rapidly growing amount of publicly available proteomics data opens up the opportunity for in silico proteomics, that is using bioinformatics to test hypotheses directly through the available data, instead of going via the generation of new experimental data.

The ability to compare protein abundances among datasets across public repositories would provide the possibility to virtually create new quantitative experiments, paving the way for in silico proteomics (Fig. 4). However, accurate absolute quantification of peptides and proteins in datasets is made challenging by the need for internal standards. Relative quantification is impaired by the heterogeneity of the data present in repositories, and their often suboptimal annotation 118. It is therefore worth mentioning that in‐depth annotation of the experimental design is essential in order to correctly interpret quantitative information from public proteomics data.

The development of bioinformatics and statistics tools for the robust and accurate interpretation of such heterogeneous data will allow the setup of creative designs where datasets from different sources can be repurposed and compared. This could, for example, enable the in silico comparison of large patient cohorts based on the aggregation of multiple smaller cohorts. Such approaches can, however, be made impossible if significant sample variability is introduced during sample extraction and preparation, for example when PTM enrichment is conducted.

## 2 Discussion

The growing amount of publicly available proteomics data has already been put to great use, both as a means to validate published results and to generate new knowledge via reprocessing and repurposing. With the achievement of the required milestones for data sharing (i.e. data standards, user‐friendly software, and public databases) and the push toward public data from journals and funders, the amount of shared data will only continue to grow rapidly.

There are, however, still some limiting factors that ought to be addressed. The first of these is the need for proper annotation, especially regarding experimental design. Indeed, even though minimal reporting standards have been developed for proteomics data (the so‐called MIAPE (Minimum Information About a Proteomics Experiment) guidelines 119), there remains a gap between what is reported and what ought to be reported. While it is possible to attempt to infer the missing information as, for example, done by the pride‐asap pipeline 120, this is often far from straightforward and may result in incorrect assumptions. The only real solution is to make it easier for submitters to provide additional information, or to annotate this information automatically in the standard file formats. This work has already started, notable in LIMS systems such as MASPECTRAS 121, ms_lims 122, and Proteios 123, but it will still take some time before it is straightforward to capture all the desired information.

A related challenge is the provision of easy access to public data while catering to the need for visual and interactive analysis 124. There are already several tools, including PRIDE Inspector 27 and PeptideShaker 72 that support this concept, but more are certainly needed. This is especially true for tools that link and display information from multiple resources in a meaningful way. Easy access for developers is also vital, for example, via systems such as BioMart 125, or more recently, via web services 48, 49.

It is also crucial that scientists get credit for sharing their data, especially when these data are reused in new contexts. The ProteomeXchange accession number should therefore always be used when a dataset is reused and the corresponding publication(s) should be cited. ProteomeXchange also issues a DOI (Digital Object Identifier) to “Complete” submissions (i.e. submissions where data are provided in accordance with public standards, so they are easier to access and reuse), as a way to improve dataset tracking and to give credit to authors 126. It will also be useful if resources provide dataset access statistics, given the current trend of putting increased value on so‐called “altmetrics” methods 127 to capture the impact of scientists’ work.

Moving forward, data‐independent acquisition approaches such as MS^E^ and SWATH‐MS will become more popular in the field 128. And even though some public data for these approaches already exist, it is expected that public deposition of this type of data will significantly increase in the coming years. In fact, there are already dedicated resources in place such as SWATH‐Atlas (http://www.swathatlas.org) that can be used for planning SWATH experiments, for depositing experiments, and for exploring the results of deposited datasets. A particular characteristic of SWATH‐MS data is that, once generated, these can potentially be reanalyzed multiple times using different spectral libraries, which are set to improve over time as public data increase. These developments open up numerous novel possibilities for the reanalysis of public proteomics data.

Another very interesting upcoming opportunity is the reprocessing of datasets generated in “multi‐omics” studies. At present, these type of studies pose a challenge for both traditional repositories, which are most often field‐specific (e.g. proteomics, genomics, or transcriptomics), as well as for researchers, given that at present it is not straightforward to link public data coming from paired samples located in different resources (e.g. MS proteomics and RNAseq data obtained in the same study). There are, however, ongoing efforts to link different studies performed on the same sample 129. Over time, the existence of personalized sequence databases (from DNA exome sequencing), or the existence of public data containing both gene and protein expression data for a given sample will become commonplace, opening up yet more opportunities for data analysts.

Many of the approaches highlighted in this review can also be exploited in the metabolomics field, where the first stable data repositories and data standards are now starting to be established 130. For example, spectral libraries have been used for the analysis of MS metabolomics data already, many years before the same approach was applied to the proteomics field, and we can expect to see more examples of techniques adopted from related fields in the future.

Finally, the need for customizable, large‐scale reprocessing systems should be highlighted. Such capabilities currently remain limited to a couple of dedicated proteomics bioinformatics groups. However, as the data have been generated by the community, and thus belong to the community as a whole, large‐scale reprocessing should also be made available to the general community. Only then can we start to realize the full potential of the publicly shared proteomics data.

*The authors have declared no conflict of interest*.
